# The reliability of spinal motion palpation determination of the location of the stiffest spinal site is influenced by confidence ratings: a secondary analysis of three studies

**DOI:** 10.1186/s12998-016-0131-x

**Published:** 2016-12-20

**Authors:** Robert Cooperstein, Morgan Young

**Affiliations:** Palmer College of Chiropractic, San Jose, 90 East Tasman Drive, San Jose, CA 94577 USA

**Keywords:** Spinal motion palpation, Interexaminer reliability, Spine, Fixation, Spinal stiffness assessment, Measurement error, Cervical, Thoracic, Lumbar

## Abstract

**Background:**

This is a secondary analysis of three previous studies on the cervical, thoracic, and lumbar spines. It uses continuous analysis of the stiffest spinal site rather than more typical level-by-level analysis to assess interexaminer reliability, and the impacts of examiner confidence and spinal region. The primary goal was secondary analysis of the combined data; secondary goal was de novo analysis of combined data emphasizing absolute indices of examiner agreement; and tertiary goal was analysis of actual vs. simulated data to determine to what degree the information provided by motion palpation impacted interexaminer reliability.

**Methods:**

This study emphasized Median Absolute Examiner Differences and Bland-Altman Limits of Agreement to calculate examiner differences, which are immune to subject homogeneity, and de-emphasized intraclass correlation, which is not. It calculated Median Absolute Deviation to determine data dispersion. The study analyzed the entire *n* = 113 combined dataset, as well as subgroups stratified by examiner confidence and spinal region. Simulations were run using a random number generator to provide chance data for examiners' findings of the stiffest spinal site, the analysis of which was compared with that of the actual data.

**Results:**

Median Absolute Examiner Differences for the combined dataset were 0.7 of one vertebral level, suggesting examiners usually agreed on the stiffest spinal site or the motion segment including it. When both examiners were confident in their findings (53.4%), the median examiner difference decreased to 0.6 levels, increasing to 1.0 levels when one lacked confidence and to 1.8 levels when both lacked confidence. Reliability was greater in the cervical and lumbar spines (each 0.6 vertebral levels examiner differences) than in the thoracic spine (1.1 levels examiner differences). The actual information provided by motion palpation compared to simulated data improved interexaminer reliability by a factor ranging from 1.8 times to 4.7 times, depending on the regional subset analyzed.

**Conclusions:**

Examiner decisions regarding the location of the stiffest spinal site were deemed adequately reliable, especially when the examiners were confident. Researchers and clinicians alike might best design their study protocols and practice methods using the stiffest segment protocol as an alternative to level-by-level spinal analysis.

## Background

Motion Palpation (MP) of the spine is integral to practitioners of manual therapy, despite the fact that most studies show it to be unreliable, with interexaminer reliability usually found near chance levels of agreement [1–4]. What may be the most complete systematic review of MP retrieved 44 articles, among which only eight reported relatively high levels of reproducibility; only four of these were judged to be of acceptable quality [5]. Possible explanations for the low reliability of MP have invoked variation in procedure [6], poor interexaminer numeration of spinal levels [7, 8], inaccurate determination of spinal landmarks [9–11], and variations in patient anatomy [12]. The fact that previous studies did not allow examiners to identify different degrees of spinal level stiffness most likely also lowered reported levels of agreement, since the likelihood of good agreement would be expected to diminish if one or both of the examiners did not find the subject to exhibit significant spinal level stiffness. Low indices of interexaminer agreement pose a threat to the clinical utility of MP, since a patient assessment procedure must be both reliable and valid to be clinically useful.

**Fig. 1:**
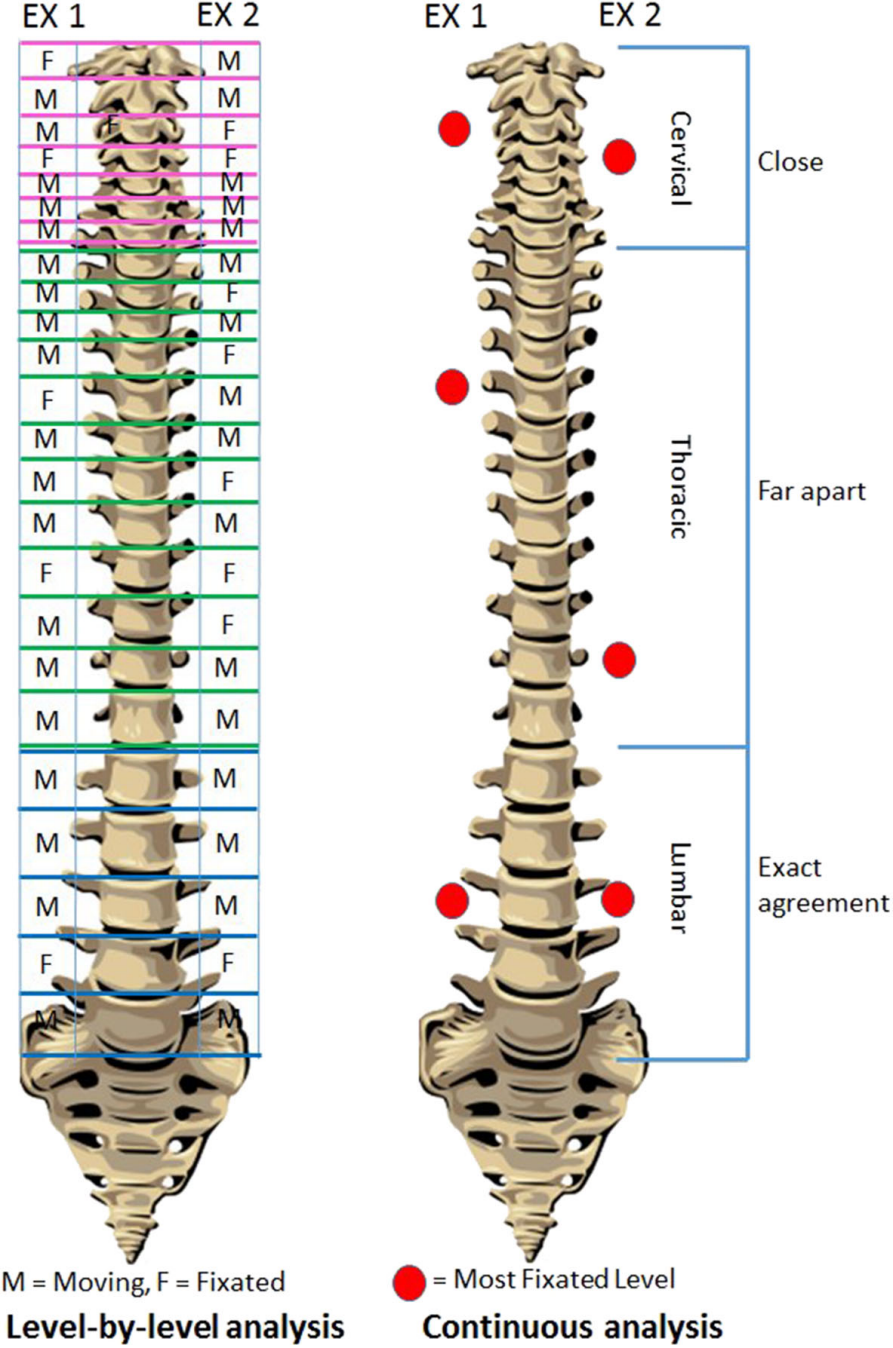
In level-by-level analysis (*left*) each examiner rates each vertebra or motion segment as judged as “moving” or “not moving”. Using continuous analysis (*right*) the examiners’ findings for the stiffest spinal site are found to be relatively close or further apart

The author and his co-investigators had previously performed a series of three MP studies predicated on a less commonly used method of defining and detecting interexaminer agreement. Instead of asking the examiners to rate individual spinal levels as exhibiting or not exhibiting stiffness to palpation, they were asked to identify the location within a defined spinal range that constituted the “stiffest spinal site” (SSS). Figure 1 depicts two contrasting methodologies that may be used to study the reliability of MP: level by level evaluation requiring level-by-level (discrete) analysis, and the SSS paradigm, which is amenable to continuous analysis. In addition to deploying an SSS paradigm, these three MP studies differed from the great majority of prior studies by allowing the examiners’ findings and the statistical analysis to be stratified by degree of examiner confidence. Examiner confidence might best be understood as a surrogate measure for the degree of spinal stiffness. Using these methods, the study team was able to demonstrate high levels of interexaminer agreement in separate studies of the thoracic [13], cervical [14], and lumbar [15] spines, published in that order. As the most representative measure, the Mean Absolute Examiner Differences in identifying the location of the stiffest spinal site, when both examiners were confident, were 2.0 cm for the thoracic spine, 1.2 cm for the cervical spine, and 2.4 cm for the lumbar spine. These examiner differences were ≤ the length of one vertebral segment: 2.3 cm for a typical thoracic segment [16], 1.8 cm for a typical cervical segment [17], and 4 cm for a typical lumbar segment [18].

In explaining these results, which reported substantial interexaminer reliability despite the dismal results of the great majority of prior studies, the authors did not invoke improvements in palpatory technique per se. To explain the improvement, the authors opined that substantial interexaminer agreement improvement could be detected by using continuous measurement of the location of the stiffest spinal site, especially when the examiners were confident in their findings; whereas more typical level-by-level analysis of agreement at each spinal level, unstratified by examiner confidence, apparently does not allow adequate reliability in identifying the stiffness of specific levels. A case was made for recording data continuously and analyzing them with appropriate statistical measures for continuous data.

The primary objective of the present study was secondary analysis of combined data from the previously published MP studies [13–15] to determine the interexaminer reliability of determining the location of the stiffest spinal site using Mean and Median Absolute Examiner Differences, Intraclass Correlation, and Bland-Altman Limits of Agreement (LOA). (Bland-Altman LOA are denoted as LOA for the rest of this article.) The secondary objective was to investigate the impact of the examiners’ confidence in their findings on these indices of reliability. The tertiary objective was to determine the degree to which actual examiner findings improved interexaminer reliability compared with random computer-generated examiner findings.

## Methods

The raw data were abstracted from previously published studies on the interexaminer reliability of thoracic [13], cervical [14], and lumbar [15] MP, and the indices of agreement featured in these studies are reported (Table 1). In each of these, examiners were asked to state whether they were confident or not confident in their determination of the SSS. If “confident”, the examiner was reasonably certain he had identified a spinal level stiffer than any other in the spinal region of interest. Therefore, the degree of examiner confidence was in part a surrogate for estimating the degree of fixation: from an operational definition point of view, examiners were more confident in their findings when they perceived a higher degree of fixation. Lack of examiner confidence in the examiners’ rating of the SSS might have come about in two different ways: an examiner might not have found *any* segment significantly stiff to palpatory pressure, or an examiner may have found multiple segments significantly but indistinguishably stiff to palpatory pressure. In the cervical study, the SSS was sought in the range C1-7, in the thoracic study T3-11, and in the lumbar study L1-5.

**Table 1:** Indices of interexaminer reliability and sample sizes in three previously published studies and in present secondary (combined) analysis

Study	*N*	ICC (2,1)	MeanAED	MedAED	LOA	RMSE	MSE
Thoracic	52	Y	Y	N	N	N	N
Cervical	27	Y	Y	N	N	Y	Y
Lumbar	34	Y	Y	Y	Y	N	N
Combined	113	Y	N	Y	Y	N	N

*Abbreviations*: *ICC* intraclass correlation, *MeanAED* Mean Absolute Examiner Difference, *MedAED* Median Absolute Examiner Difference, *LOA* limits of agreement, *RMSE* root mean squared error, *MSE* mean squared error

An examiner’s finding was recorded by having either a research assistant or the first examiner place a skin mark at the SSS followed by a research assistant measuring the distance in cm from that skin mark to a fixed point (S1 in the thoracic study and lumbar studies, and the vertebra prominens in the cervical study; as determined by palpation). Subtracting the second examiner’s distance from the landmark from the first examiner’s distance from the landmark provided the distance between their individual determinations of the SSS. The examiners (a) were blinded from each other’s findings; (b) were not provided any clinical information about the subjects (c) alternated their order in assessing the subjects; and (d) did not converse with the subjects. The elapsed time between examiners’ observations was 2–5 min.

The subjects were asymptomatic or minimally symptomatic. Unlike the thoracic and lumbar studies, which featured two examiners, the cervical study involved three examiners. In order to facilitate secondary analysis of all three spinal regional studies, and to avoid overweighting the cervical data, the authors included only data for examiner two vs. examiner three from the cervical study. These data for interexaminer reliability appeared to lie between those of the other two examiner comparisons. In all three prior studies the subjects were a convenience sample of students who were either asymptomatic or had spine pain ≤2 on a 0–10 scale. The examiners were all instructors at the chiropractic college and private practitioners (either past or present), two with 25–30 years of experience (RC and MH) and one with eight years (MY). The palpators in the cervical study were RC and MH, in the thoracic study RC, MH, and MY, and in the lumbar study RC and MY.

In the three original studies, interexaminer reliability was reported using a variety of indices of interexaminer reliability, as summarized in Table 1. It should be noted that the term “Mean Absolute Examiner Difference” (MeanAED), as used in the current article, was referred to as “mean of the absolute value of examiner differences” in the prior thoracic spine article, and as “absolute value examiner differences” in the prior cervical spine article. This secondary analysis calculated interexaminer reliability using the following statistical measures: Intraclass Correlation (ICC(2,1)), Standard Error of Measurement for ICC [19], Mean Absolute Examiner Difference (MeanAED), Median Absolute Examiner Difference (MedAED), and 95% LOA [20, 21]. This secondary analysis, unlike the previously published studies [13–15], combined the data in the cervical and lumbar spines for the two subsets in which at least one examiner lacked confidence: (a) one examiner not confident; and (b) both examiners not confident. This helped avoid subsets too small to meaningfully analyze. This was not deemed strictly necessary for the thoracic spine, where no subset included less than 10 subjects, nor for the combined dataset.

The interpretation of the ICC findings in this study is based on the following commonly-cited cutoffs for qualitative ratings corresponding to ICC values: interexaminer reliability is judged “poor” for values less than .40, “fair” between .40 and .59, “good” between .60 and .74, and “excellent” for values between” .75 and 1.0 [22]. The dispersion of absolute examiner differences was reported using range and, also Median Absolute Examiner Deviations (MADmedian) [23, 24]. Calculating MADmedian [23] involves (a) identifying the median value of absolute examiner differences, (b) subtracting this value from each examiner difference and converting to an absolute value; and (c) calculating the median of this derived set of values. Analyses were conducted stratified by both spinal region and examiner confidence. Shapiro-Wilk testing was conducted to confirm the normality of the distributions of examiner differences, since this property is required to support the use of some of the statistics used in this study (LOA, ICC).

In addition to being provided in cm units, MeanAED, MedAED, MADmedian range estimates, and LOA were transformed into and presented as vertebral equivalents (VE), where VE is defined as the height of a typical vertebra. Since the height of a typical vertebra varies according to the spinal region, an examiner difference reported in cm and analyzed as such would imply varying degrees of examiner reliability depending on the spinal region and the height of a typical vertebra in that region. Reporting the data as VEs is more clinically relevant and allows immediate comparisons of examiner reliability irrespective of spinal region. To convert cm to VE, the following heuristic weighting factors were used: 2.3 cm for a typical thoracic segment [16], 1.8 cm for a typical cervical segment [17], and 4 cm for a typical lumbar segment [18].

In addition to analyzing the actual study data, a series of simulations were run to provide chance data for examiners’ findings of the SSS. To do so, for each spinal region a random number generator created a series of n paired numbers (representing simulated examiner findings for the SSS) ranging from 0.00 to 0.99, where n equals the sample sizes used in the actual study. Then, the absolute value of the differences in the paired values, in effect a fraction of the length of the relevant spinal region (20.0 cm, 20.7 cm, and 12.6 cm for the lumbar, thoracic and cervical spinal regions respectively) was multiplied by the range in cm of the related spinal region to produce a series of simulated examiner difference in cm, which was in turned converted to VE units. This procedure enabled determining to what extent the information provided by MP impacted interexaminer reliability.

## Results

**Fig. 2:**
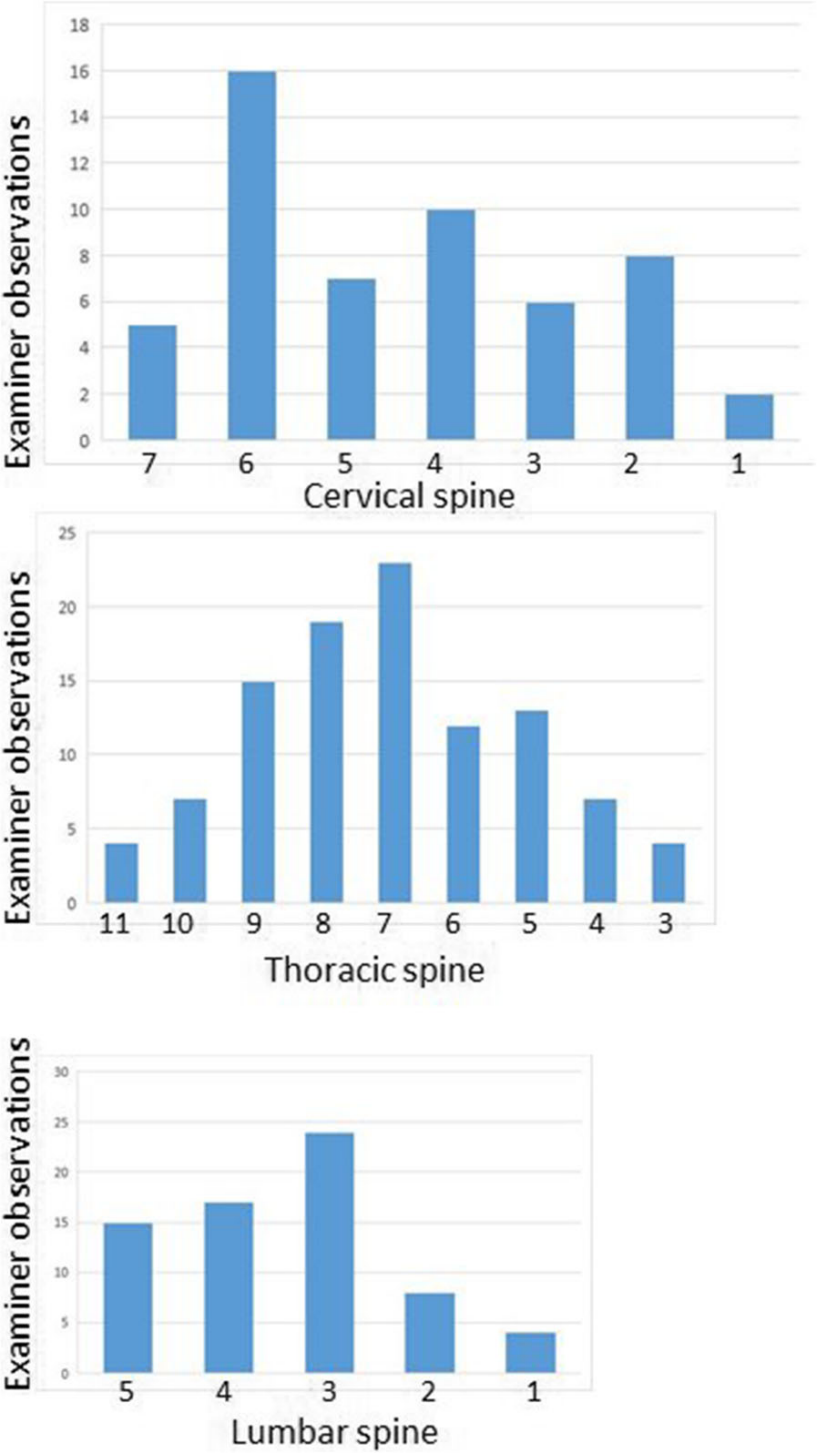
Number of examiner observations for SSS at each spinal level

The demographic data for the included studies are reported (Table 2). The distribution of examiners’ findings for the SSS are shown in Fig. 2: the most frequent finding for the SSS was C6 in the cervical spine, T7 in the thoracic spine T7, and L3 in the lumbar spine. Among 226 patient assessments (113 subjects each assessed by two examiners), an examiner was confident 158 times (69.9%). Both examiners were confident 61 times (53.4% of subjects); only one examiner was confident 36 times (31.9% of subjects); and neither examiner was confident 16 times (14.2% of subjects).

**Table 2:** Demographics of the participants in the three original studies and the combined sample

Study	*N*	Age (years)	Gender, % female	Pain (0–10)
Thoracic	52	25.8	21.2	0.7
Cervical	27	27.1	35.5	0.8
Lumbar	34	25.4	54.3	0.5

The values of ICC(2,1) and MeanAED for all three spinal regions, stratified by confidence, in both cm and VE units, are reported (Table 3). Although to be thorough the data are reported in both cm and VE, the interpretation of results stresses the latter. Among 10 ICC(2,1) values reported, only one was judged “excellent”: both examiners confident, thoracic spine. Two were judged “good”: both examiners confident in the cervical spine; and for all subjects in the cervical spine. For the combined dataset, it was not possible to calculate ICC values nor deemed meaningful to calculate mean examiner differences in cm; hence the relevant cells were left deliberately blank in Table 3. Calculating an ICC for all 113 observations would have been inappropriate because the examiners in the cervical study, for example, were constrained to find the SSS among only seven vertebrae rather than among all 24 vertebrae in the spine; likewise, in the thoracic and lumbar studies, where examiners were constrained to examine only 9 and 5 vertebrae, respectively. Calculating ICC for the combined dataset would have exaggerated the degree of reliability.

**Table 3:** Mean Absolute Examiner Differences (cm and VE), and associated ICC (2,1) values

*N*	Subset, *N*	MeanAED, cm	Range, cm	MeanAED, VE	Range, VE	ICC(2,1)	SEM (cm)	Rating
Cervical spine								
27	All subjects	1.3	0.0–3.2	0.7	0.0–1.8	0.60 (0.30, 0.80)	1.1	Good
21	Both examiners confident	1.2	0.0–3.2	0.7	0.0–1.8	0.66 (0.35, 0.85)	1.0	Good
6	≥1 examiner not confident	1.8	0.8–2.6	1.0	0.4–1.4	0.42 (0.00, 0.89)^a^	1.3	Fair
Thoracic spine								
52	All subjects	4.0	0.0–14.8	1.7	0.0–6.4	0.31 (0.05, 0.54)	3.9	Poor
21	Both examiners confident	2.0	0.2–7.5	0.9	0.1–3.3	0.83 (0.63, 0.93	1.9	Excellent
21	One examiner not confident	4.5	0.0–14.8	2.0	0.0–6.4	0.00 (0.00, 0.28)^a^	4.3	Poor
10	No examiners confident	7.1	1.4–14.6	3.1	0.6–6.3	0.00 (0.00, 0.28)^a^	4.3	Poor
Lumbar spine								
34	All subjects	2.6	0.2–7.1	0.7	0.1–1.8	0.39 (0.06, 0.64)	2.3	Poor
19	Both examiners confident	2.4	0.2–7.0	0.6	0.1–1.8	0.09 (0.00, 0.52)^a^	2.2	Poor
15	≥1 examiner not confident	2.9	0.0–7.1	0.7	0.0–1.8	0.52 (0.03, 0.81)	2.5	Fair
Combined dataset								
113	All subjects	Intentionally blank		1.2	0.0–6.4	Intentionally blank		
61	Both examiners confident			0.7	0.1–1.8			
36	1 examiner confident			1.5	0.0–6.4			
16	No examiners confident			2.1	0.0–6.3			

*Abbreviations*: *MeanAED* Mean Absolute Examiner Differences, *VE* vertebral equivalent, *ICC* intraclass correlation, *SEM* standard error of measurement

^a^Negative ICC value reported as 0.00

Unstratified MeanAED in the cervical spine and lumbar spine was 0.7 VE, suggesting average examiner agreement on the motion segment including the SSS. Unstratified MeanAED in the thoracic spine was 1.7 VE, suggesting examiner agreement on either the same or two adjacent motion segments including the SSS. Unstratified MeanAED in the combined dataset was 1.2 VE, suggesting examiner agreement on the motion segment including the SSS. When both examiners were confident, for all three spinal subsets and for the combined data, MeanAED ≤0.9 VE, consistent with examiner agreement on the motion segment including the SSS. In each subset, examiner differences decreased when examiner confidence increased.

The analogous MedAED results are also provided (Table 4), including information on the *dispersion* of examiner differences, MADmedian. The *median* examiner differences in Table 4 are similar to the *mean* examiner differences in Table 3 (as one would expect given that the mean and median values of quasi-normal distributions tend to be similar), but show across the board smaller examiner differences, since median calculations are insensitive to extreme outliers. Given all unstratified MedianAED ≤ 1.1 VE, the data are consistent with average examiner agreement on the motion segment above or below the SSS. The median unstratified examiner difference for the combined dataset was relatively small, 0.7 VE (Table 4). This decreased to 0.6 VE when both examiners were confident, a little more than half a vertebral height. Since MedAED for the combined dataset was 0.7 VE and MADmedian was 0.5VE, it may be concluded that 50% of examiner differences were 0.7 ± ≤0.5 VE, thus between 0.2 VE and 1.2 VE. Had the data been normally distributed, 75% of the examiner differences would have been ≤1.2 VE. Due to a slight skewing of the data, in fact 73.4% of examiner differences were below 1.2 VE.

**Table 4:** Median Absolute Examiner Differences and Median Absolute Deviations (cm and VE)

*N*	Subset	MedianAED, cm	MADmedian, cm	MedianAED, VE	MADmedian, VE
Cervical					
27	All subjects	1.1	0.7	0.6	0.4
21	Both examiners confident	1.0	1.0	0.6	0.6
6	≥1 examiner not confident	1.7	0.7	0.9	0.4
Thoracic					
52	All subjects	2.5	2.0	1.1	0.9
21	Both examiners confident	1.5	1.0	0.7	0.4
21	One examiner not confident	3.0	2.4	1.3	1.0
10	No examiners confident	5.0	2.0	2.2	0.9
Lumbar					
34	All subjects	2.5	1.8	0.6	0.4
19	Both examiners confident	2.3	1.7	0.6	0.4
15	≥1 examiner not confident	1.7	0.7	0.4	0.2
Combined dataset					
113	All subjects	2.1	1.4	0.7	0.5
61	Both examiners confident	1.4	0.9	0.6	0.4
36	1 examiner confident	2.9	1.8	1.0	0.5
16	No examiners confident	4.1	2.8	1.8	1.1

*Abbreviations*: *MedAED* Median Absolute Examiner Difference, *MADmedian* Median Absolute Deviation, *VE* vertebral equivalent

The LOA [20, 21] for interexaminer agreement, in both cm and VE, are provided (Table 5). The column for fixed bias, the simple average of examiner differences, shows very small systematic differences (<½ vertebral height for each spinal region) in the examiners’ assessment of the SSS in all the subsets. Histograms of examiner differences (not shown) confirmed these differences were normally distributed, satisfying the requirement for calculating LOA that examiner differences come from a normal population. The 95% LOA for examiner differences when both examiners were confident for the SSS were ±1.5 VE in the cervical spine, 2.6 VE in the thoracic spine, 1.6 VE in the lumbar spine, and 1.9 VE for the combined dataset. It must be emphasized that these LOA do not identify the *mean* examiner differences, but rather the *boundaries* that contain 95% of examiner differences. Analysis of the LOA for the other subsets confirms that increasing examiner confidence decreased the LOA. For example, when neither examiner was confident in the combined dataset, the 95% LOA was 5.5 VE, which is 2.8 times wider than the 95% LOA when both were confident.

**Table 5:** Bland-Altman Limits of Agreement (cm and VE) for examiner determination of SSS

				95% LOA	
*N*	Subset	Units	Bias	Lower limit	Upper limit
Cervical spine					
27	All subjects	cm	0.0	−3.1	3.1
		VE	0.0	−1.7	1.7
21	Both examiners confident	cm	−0.2	−3.1	2.7
		VE	−0.1	−1.7	1.5
6	≥1 examiners not confident	cm	0.7	−3.2	4.5
		VE	0.4	−1.8	2.5
Thoracic spine					
52	All subjects	cm	−0.1	−10.9	10.8
		VE	0.0	−4.8	4.7
21	Both examiners confident	cm	0.6	−4.8	6
		VE	0.3	−2.1	2.6
31	≥1 examiners not confident	cm	−0.5	−13.9	12.9
		VE	−0.2	−6.1	5.6
Lumbar spine					
34	All subjects	cm	0.5	−6.1	7.1
		VE	0.1	−1.5	1.8
19	Both examiners confident	cm	0.3	−5.9	6.5
		VE	0.1	−1.5	1.6
15	≥1 examiners not confident	cm	0.8	−6.4	7.9
		VE	0.2	−1.6	2.0
Combined dataset					
113	All subject	cm	0.1	−8.2	8.4
		VE	0.1	−3.0	3.1
61	Both examiners confident	cm	0.3	−4.7	5.2
		VE	0.1	−1.7	1.9
36	1 examiner confident	cm	0.9	−10.8	9.1
		VE	−0.3	−4.0	3.3
16	No examiners confident	cm	1.90	−10.9	14.7
		VE	−0.7	−4.0	5.4

*Abbreviations*: *LOA* limits of agreement, *SD* standard deviations, *SE* standard error, *VE* vertebral equivalent

**Fig. 3:**
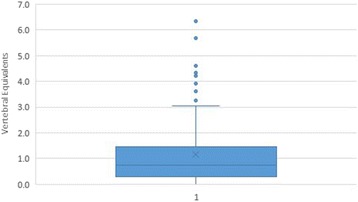
Box-and-whisker plot for examiner differences, measured in VE units, combined dataset

A box-and-whisker plot is provided to summarize the results in the combined dataset (Fig. 3). The plot divides the data into 4 equal parts. The low whisker represents the bottom 25% of examiner differences, those that were smallest (measured in VEs); the box represents the middle half of the examiner differences; and the upper whisker the top 25%, the largest examiner differences. The dots outside the whiskers represent those data points that are considered outliers, defined as such because they are out of the box and beyond the third quartile of the data by more than 1.5 times the interquartile range, or height of the box. Analysis of the plot leads to the conclusion that 88/113 (77.0%) of examiner differences were ≤1.5 VE, and 10/113 (8.8%) of examiner differences were ≥1.5 times the interquartile range, extreme data points generally considered outliers [25].

MedAED calculations using randomly generated on the one hand were contrasted with calculations based on the actual data on the other hand (Table 6). For the cervical spine, chance alone resulted in MedAED = 2.9 VE, whereas in fact the actual unstratified MedAED = 0.6 VE; reliability using real clinical data exceeded reliability using chance data by a factor of 4.7 times. In the thoracic spine, chance MedAED = 2.0 VE, whereas the real MedAED = 1.1 VE, resulting in 1.8 times better reliability. In the lumbar spine, chance MedAED = 2.4 VE, whereas the real MedAED = 0.6 VE, resulting in 3.8 times better reliability. In the combined dataset, including data from all three spinal regions, chance MedAED = 1.9 VE, whereas the real MedAED = 0.7 VE, resulting in 2.5 times greater reliability.

**Table 6:** Simulations based on data for Average Examiner Differences (VE) created using a random number generator

Study	*N*	Actual data, VE	Simulated data VE	Simulated/Actual (% improvement)
Cervical	27	0.6	2.9	470%
Thoracic	52	1.1	2.0	180%
Lumbar	34	0.6	2.4	380%
All	113	0.7	1.9	250%

## Discussion

Among four dozen MP studies included in an annotated review of MP reliability studies [1], Potter et al. [26] were the only investigators to have used a SSS method and ICC analysis similar to the present included studies. Since theirs was an *intraexaminer* study, unlike the present study, and furthermore included postural and movement asymmetry in the examination panel in addition to MP, the results of their study cannot be directly compared with the present results. The palpators in the present study did not verbally interact with the subjects, ensuring that the findings of spinal level stiffness alone were central to the identification of dysfunctional spinal segments, not confounded by subjective information concerning pain or tenderness.

Broadly speaking, subset analysis in Tables 3, 4 and 5 supports each of the following statements: (a) increased examiner confidence was associated with increased interexaminer reliability; (b) interexaminer reliability was greater in the cervical and lumbar spines than in the thoracic spine; and (c) examiner confidence had a more variable impact on examiner agreement in the regional analyses than in the whole dataset. These trends are especially visible in the data for the thoracic spine and for the combined dataset. In the thoracic spine, MedAED was 0.7 VE when both examiners were confident, but increased to 1.3 VE when at least one examiner lacked confidence and to 2.2 VE when both lacked confidence. In the combined dataset, MedAED was 0.6 VE when both examiners were confident, increasing to 1.0 VE when one examiner lacked confidence, and to 1.8 VE when neither was confident.

The subjects were relatively homogeneous in their SSSs (Fig. 2), with the most frequently identified SSS for the cervical spine being C6, the thoracic spine T7, and the lumbar spine L3. ICC is not the ideal index of interexaminer reliability when, as in our studies, the subjects are relatively homogeneous. In that circumstance, ICC becomes misleadingly low [27]. This results from the fact that ICC is a ratio of the variance within subjects to the total variance (the sum of within-subject and between-subjects variance). When between-subjects variance is relatively low, the ICC level diminishes even when and if the examiners largely agree. To illustrate the fact that ICC is very sensitive to subject homogeneity, the previously published lumbar study [15] constructed a hypothetical dataset in which examiner differences were equal to those seen in the actual dataset, but in which the findings of the SSS were more evenly distributed across the lumbar spine. In this hypothetical dataset, ICC(2,1) increased from 0.39 (“poor”) in the real dataset to 0.70 (“good”) in the hypothetical dataset, despite examiner differences being equal, subject for subject, in the two datasets.

To offset the interpretation of the misleadingly low ICC values in this study, the authors emphasized indices of interexaminer reliability that were immune to it: MeanAED, MedAED, and LOA [20, 21]. MedianAED calculations are especially preferred [23, 24] because they are immune to the impact of extreme values [23], which do conversely impact the calculation of MeanAED and LOA. From a clinician’s point of view, it ought to be intuitively obvious that the happenstance of occasional large differences in two examiners’ determination of the SSS ought not distract them from the clinical utility of an examination protocol using which *usually* results in agreement on the SSS or the motion segment including it. These MedianAED calculations reinforce confidence in the protocol. The insensitivity of median calculations to extreme values accounts for why the MedianAED values were generally smaller than the MeanAED values in this study. Although either MedAED or LOA calculations may have sufficed unto itself, it was deemed more convincing to deploy each to check for consistency between methods. Between the two, the LOA are more conservative estimates of examiner agreement, as explained below.

Interpretation of the subsets in Tables 3 and 4, which are stratified by spinal region and examiner confidence, becomes misleading as the size of the subsets diminished. When a sample size is small, the results of the analysis can be altered considerably by shifting a very small number of data points from one clinical result to another. Walsh [28] has described a Fragility Index: “the minimum number of patients whose status would have to change from a nonevent to an event to turn a statistically significant result to a non-significant result.” As an example using the lumbar ICC values, if the two examiners had exactly agreed on subject 13, rather than disagreed by 7.1 cm (the largest disagreement in this subset), the ICC(2,1) for all subjects in the lumbar subset would have increased from the reported 0.39 to 0.46, and the interpretation would have changed from “poor” to “fair.” Likewise, if the two examiners had disagreed on subject 32 by 7.1 cm rather than exactly agreeing, the ICC(2,1) for the *N* = 15 subset where at least one examiner lacked confidence would have decreased from 0.52 to 0.43. Therefore, shifting only two of 34 data points would have negated the otherwise paradoxical finding in the actual lumbar study that less confidence in the lumbar spine was associated with *smaller* examiner differences.

The columns labeled MAD in Table 4 represent the degree of data dispersion, how spread out the data are. It paints a more complete picture than the more typically reported *range*, the simplest measure of dispersion, the difference between the maximum and minimum values. The primary problem with reporting simple range is that it is very impacted by extreme minimum or maximum values. Standard deviation and variance, although very widely used to assess dispersion in normal distributions, are also impacted by outliers, since a data point very distant from the others can substantially increase their computed values. In addition, when using standard deviation as a measure of data dispersion, the distances from the mean are squared, so large deviations are weighted more heavily. MADmedian is robust to such extreme values (i.e., it is not impacted by them), since a larger extreme value has no greater impact than a smaller extreme value. The primary strength of MADmedian is also an important weakness: so-called extreme outliers at the lower and upper quartiles of examiner differences may represent an important characteristic of the examination method under investigation.

**Fig. 4:**
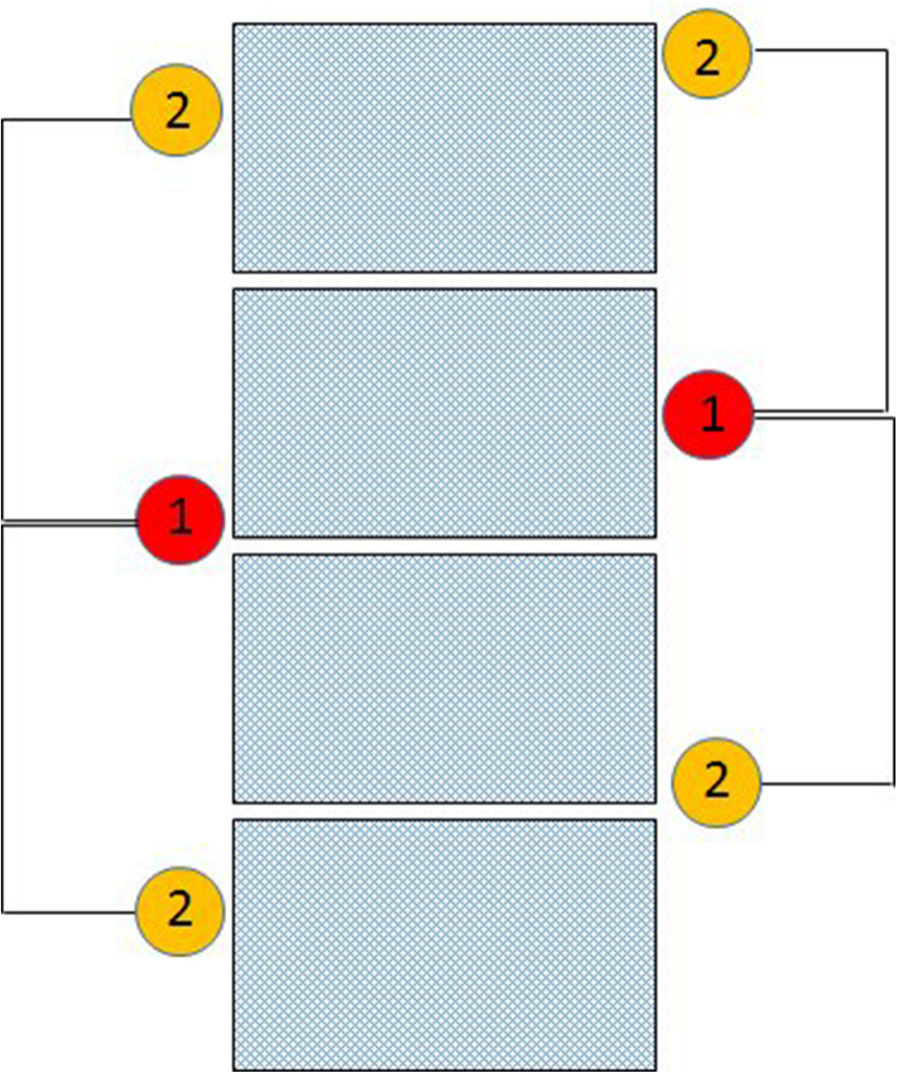
If examiner 1 finds the SSS at bottom or top of vertebra (*left*), 1.5VE difference for examiner 2 suggests agreement on either the motion segment above *or* the *adjacent* motion segment below. If examiner 1 finds the SSS near middle of vertebra (*right*), 1.5VE difference for examiner suggests agreement on either the motion segment above or below

To assist in interpreting the findings for MedianAED (Fig. 4), let us consider a case in which the first examiner has judged the SSS to be at the exact middle of a given segment. So long as the second examiner identifies a SSS that is not more than 1.5 segments away, it can be stated they at least agreed on the motion segment including the SSS, and may have agreed on the SSS itself. Given the findings of the first examiner, this agreement may have occurred on the motion segment above or below. That stated, we must be careful to make clear this does not imply their findings were somehow in a range spanning 2 motion segments. It simply means in some cases they agreed on the motion segment above that identified by examiner 1, and in other cases below. If, on the other hand, an examiner had identified the SSS at the most inferior or superior aspect of a given segment, the other examiner must not have been disagreed by more than 1.0 VE for them to have identified the same segment or the motion segment containing it. This happened 60.2% of the time. In this study, as can be seen in the box-and-whisker plot (Fig. 3), examiner differences were ≤1.5 VE 77.0% of the time in the combined dataset. It would be very difficult, if not impossible, to tease more accurate numbers from these studies, so as to know whether the frequency of missing by more than one motion segment is closer to 23.0 or 39.8%. Doing so would require untenable assumptions as to exactly how the location of what the examiners actually touched in these 3 different anatomical regions (the articular pillar in the cervical spine, transverse processes in the thoracic spine, and spinous process in the lumbar spine) related to the actual center of the vertebrae.

When both examiners were confident, their differences were ≤1.0 VE in 55 of 61 (90.2%) of cases, which is to say they definitely agreed on the motion segment containing the SSS 9 (again, on the motion segment above or below given the findings of the first examiner); and in only one case (1.7%) did they differ by more than 1.5 VE, suggesting they definitely disagreed on the motion segment containing the SSS. When one of the examiners lacked confidence, their differences were ≤1.0 VE in 18 of 36 (50.0%) of cases; when neither examiner was confident, there were no cases when their difference was ≤1.0VE. Outliers, defined as such because they were ≥1.5 times the interquartile range, may have occurred when a subject had more than one spinal segment that was stiff in the range being examined, and yet the examiner was constrained to decide upon the stiffest segment.

The 95% LOA round off to −3.0, 3.1 VE. This may be interpreted as follows: 95% of examiners’ differences for the SSS were ≤ 3 vertebral heights apart. It must be emphasized that these LOA do not identify the *mean* examiner difference, but rather the *boundaries* that contain 95% of examiner differences. Increasing examiner confidence decreased the LOA; when both examiners were confident, 95% of the time they were ≤ 1.8 levels apart. The LOA were smaller in the lumbar and cervical spines, but relatively larger in the thoracic spine, presumably due to its greater length. Identifying the stiffest spinal site among nine in the thoracic spine might have resulted in relatively lower agreement compared with identifying it among only five vertebrae in the lumbar spine. With more choices available, there is a greater risk of finding two or more levels stiff. In our forced-call method, where the examiners had to choose the stiffest segment, palpators who largely agreed on those two segments might have disagreed as to which was stiffest.

Since LOA are derived using calculations that involve squaring examiner differences, they generally result in wider confidence intervals for examiner differences than the ranges established by MedAED calculations. Therefore, it may be said they are more conservative in their estimation of interexaminer agreement. The choice between using less and more conservative measures of examiner agreement might best depend on the clinical significance of the measurements. For example, if two technologies for measuring a lab value obtain measures on opposite sides of a benchmark number supporting or not supporting prescribing a medication, the safety of a patient may be compromised depending on which technology is emphasized. However, in performing motion palpation for spinal stiffness, there is little if any evidence that examiner differences in judgement are likely to significantly compromise the health status of the patient.

Table 6, which compares interexaminer reliability using randomly created chance data to the reliability that was obtained using the real data, best illustrates to what extent the information provided by MP impacted interexaminer reliability. The furthest right column provides the ratio of simulated to actual MedAED. The information improved interexaminer reliability by a factor ranging from 1.8 times to 4.7 times, depending on the regional subset analyzed. These data provide convincing evidence that MP for the SSS improves interexaminer agreement on the site of potential spine care, despite previously reported data based on level-by-level analysis that MP infrequently achieve reliability above chance levels [1–4]. There is no obvious way to compare these heuristic calculations of the enhancement of interexaminer reliability afforded by the SSS protocol with other measures of reliability that have been deemed acceptable. What defines an acceptable level of reliability for a spinal assessment procedure depends on the consequence of a mistake being made. One would suppose that a mistake on the SSS would not matter nearly as much as, for example, a mistake made by a spine surgeon concerning the intervertebral disc level thought responsible for lumbar radiculitis.

Examiners could agree on the SSS and yet both be incorrect in their determinations, as might be determined by comparison with a valid reference standard. Moreover, even were they accurate, the information might prove to be of little clinical utility. An innovative efficacy study [29] using a randomized trial study design explored whether the data provided by MP was associated with a clinically relevant pain reduction in one session of cervical manipulation compared with non-specific cervical manipulation. Although the study found endplay assessment did not contribute to same-day clinical improvement in the cervical spine, the investigators did not rule out possible contribution over a longer term.

Perfect segmental specificity on a spinal site of care is probably not strictly required, since a spinal intervention generally addresses a motion segment consisting of two vertebrae [30, 31]. As can be ascertained from both the MedAED and LOA analyses, the pairs of examiners in the three studies herein re-analyzed tended to identify the same or adjacent vertebrae as the SSS, especially when they were confident in their findings; and especially in the cervical and lumbar spinal regions.

The better reliability seen in these studies compared with the great majority of previous MP studies [1–4] is most likely not primarily attributable to improvements in the end-feel palpatory methods that were used, and may not constitute a better method for identifying the most appropriate site of spine care. The authors are not aware of any outcome studies that report different results based on characterizing every spinal level as moving or not; compared with flagging the most relevant location within a patient’s area of primary complaint. Therefore, these results do not call for clinicians to adopt new patient assessment methods nor that they change their record-keeping protocols. They do suggest that researchers might consider designing study protocols and research methods to explore reliability using the “most clinically relevant spinal site” protocol that some clinicians no doubt use, as an alternative to level-by-level analysis. In fact, these results raise the possibility that the present inventory of mostly level-by-level (certainly for MP) reliability studies may have underestimated clinically relevant examiner agreement, thereby unduly discouraging further research and clinician interest in such research. It may be possible to apply the continuous analysis approach used in the present study to other types of interexaminer reliability scenarios, including for example thermography and leg length inequality studies. In fact, at least one study on the reliability of thermographic assessment did in part use continuous analysis [32], as did two studies on assessing leg length inequality [33, 34].) These experimental design modifications may more meaningfully assess examiner agreement than the mostly level-by-level analysis that has been used up until now.

### Limitations of method

To facilitate pooling data from all three regional studies, the authors arbitrarily included only data for examiner two vs examiner three from the cervical study, excluding the data for one vs. two and two vs. three. The authors chose to use the two vs three data because its findings for interexaminer reliability appeared to lie between those of the other two examiner comparisons. Each of the prior studies included a different number of subjects; it would have been better to have equal numbers, but the subjects were recruited at different times in an environment where the size and gender mix of the convenience sample fluctuated. In the thoracic study, the range examined did not include T1-2 and T12; the investigators had formulated the clinical opinion based on prior experience that these areas were so prone to stiffness that the experimental findings of reliability could become misleadingly inflated. Among the original three included studies, only the lumbar study included a power analysis.

Some of the sample sizes in the subset analyses of the present study were clearly underpowered, suggesting caution in interpreting interexaminer reliability. The recommended number of subjects for either a complete dataset or a subset in this kind of study is about 35 subjects, to have 80% power at the 5% significance level to detect ICC ≥ 0.6 [35]. Since subsets are by definition smaller than the complete dataset, “it would be more reliable to look at the overall results of a study than the apparent effect observed within a subgroup” [36]. The subsets for one examiner lacking confidence and both examiners lacking confidence were combined in some of the analyses in this study to at least partially mitigate this effect. Although the data clearly suggested increased levels of examiner confidence bred reliability, among all the subset analyses made in this study only one reached the threshold of 35 subjects: the both examiners confident subset of the combined dataset. That stated, all of the measures of reliability in the combined dataset (MeanAED, MedianAED, and LOA) showed substantially increased reliability in the both doctors confident subset compared with the full dataset (both of which were adequate in subject size), suggesting the study’s conclusions regarding the role of confidence are reasonable.

Lacking a reference standard, it cannot be confirmed there actually were stiff spinal levels in the included studies of asymptomatic and minimally symptomatic subjects. An examiner might not have found any segment significantly stiff to palpatory pressure, or an examiner may have found multiple segments significantly but indistinguishably stiff to palpatory pressure. The study participants were largely asymptomatic, thus not reflective of symptomatic patients seeking care, jeopardizing the external validity in a manner that has been previously criticized [4, 8]. On the other hand there is some evidence that using more symptomatic participants does not appreciably change the outcome [37]. The research assistant may have introduced some error in marking and measuring the locations for each examiner’s SSS; however, the data are consistent with these putative errors having been random and thus unbiased (the bias estimates in Table 5 are all near zero). Although the examiners did not converse with the subjects, the subjects may have provided non-verbal cues such as pain withdrawal reactions or wincing gestures; these putative non-verbal cues may have impacted the examiners' findings for the SSS. To some extent this study suggested that examiner confidence breeds examiner agreement. However, since it is not known if the examiners were accurate, nothing is implied about an individual examiner’s confidence in a typical practice setting; i.e., it is not known whether it is more or less efficient to be confident in the findings of MP. The present study does not suggest that high confidence, which could very well be unwarranted by skill level, improves the *accuracy* of MP. Since this study focused on spinal hypomobility, it did not address the question of whether a putative “most hypermobile segment” might be identified using similar methods, which may arguably be quite important in clinical practice.

Although examiners agreed on the SSS or the motion segment 60.2–77.0% of the time, it is equally true that they disagreed on the SSS by more than one motion segment 23.0–39.8% of the time. Granted that clinician disagreement on the site of spinal intervention care may lead to suboptimal care or even harm patients, the authors are not aware of studies confirming or excluding that possibility. Perhaps too optimistically, but not without reason, Cooperstein and Hass wrote [38]: “Although most patients are better off after a round of chiropractic care, there are data suggesting that about half of them suffer at least one adverse consequence along the way [39, 40]. Nevertheless, these tended to be minor and transient, and we have every reason to believe that even these patients were made better off than had they received no care at all. Since most patients improve, but some more quickly and with less adverse consequences along the way, perhaps ‘wrong listings’ are not so much wrong as suboptimal. This is just what we would expect if, rather than listings being simply right or wrong, there were a listings continuum ranging from very appropriate to very inappropriate. Then listings would matter, in the sense that doing the ‘right thing’ would be better than the ‘wrong thing,’ although even the wrong thing would usually be better than literally nothing, i.e., no clinical intervention.”

## Conclusions

Neither the confidence module nor the subtyping by spinal region should obscure this study’s central finding: MP for the SSS in the combined dataset, when analyzed using continuous data and related statistical methods, is reliable and appears to identify a clinically relevant and tightly constrained location for the stiffest spinal site; and the variability of the measured interexaminer differences is low. Using a stringent criterion of agreeing on at least the motion segment including the SSS, the described continuous measures palpation protocol was reliable 60.2–77.0% of the time for the combined dataset, and was 90.2% reliable when both examiners were confident. These findings support the view of some authors who have expanded the field of examiner agreement using motion palpation to include nominated segments that are within one level of each other [30, 41]. In only 8.8% of outlier cases where examiner differences were ≥1.5 times the interquartile range were the examiners so discrepant that they must be frankly judged to have been unreliable. These findings are quite different from the very low reliability of MP that has been reported in studies that used level-by-level analysis and the Kappa statistic to report results. Readers must come to their own conclusions as to how important it is to know that spinal motion palpation, which has been widely thought to attain levels of agreement barely above chance in studies using level-by-level analysis, may now be understood based on continuous measures analysis to come to a very different conclusion: there is a 4.7-fold improvement over chance agreement in the cervical spine in identifying the location of the SSS, a 1.8-fold improvement in the thoracic spine, a 3.8-fold improvement in the lumbar spine, and a 2.5-fold improvement for the full spine.

Future researchers might consider designing study protocols and research methods to explore reliability using the “most clinically relevant spinal site” protocol as an alternative to level-by-level analysis in order to improve clinical applicability as well as reported agreement. In doing so, they might best take into account that using ICC to assess examiner agreement may understate agreement if the examiner’s findings are relatively homogeneous, clustered in narrow spinal ranges.

Beyond the issue of whether clinician error in identifying the SSS can actually harm a patient, it must be considered that such errors may result not so much in harm as reduced effectiveness in outcome studies. Finally, it should be pointed out, given the central importance of MP in virtually every institution where manual therapy is taught, this study might reassure students and practicing clinicians that under certain circumstances MP appears to be reliable notwithstanding prior research that underestimated its reproducibility.

## Abbreviations

ICC: Intraclass Correlation Coefficient
LOA: Limits of agreement
MAD: Median Absolute Deviation
MAD VE: Median Absolute Deviation expressed as vertebral height
MeanAED: Mean Absolute Examiner Difference
Med VE: Median expressed as vertebral height
MedAED: Median Absolute Examiner Difference
MP: Motion palpation
MSE: Mean squared error
RMSE: Root mean squared error
SSS: Stiffest spinal site
VE: Vertebral equivalents
